# A Method Inspired by One-Dimensional Discrete-Time Quantum Walks for Influential Node Identification

**DOI:** 10.3390/e27060634

**Published:** 2025-06-14

**Authors:** Wen Liang, Yifan Wang, Qiwei Liu, Wenbo Zhang

**Affiliations:** 1 College of Information Science and Engineering, Shenyang Ligong University, Shenyang 110159, China; 2 School of Public Administration and Policy, Dalian University of Technology, Dalian 116081, China

**Keywords:** complex networks, influential nodes, quantum computing, quantum walks

## Abstract

Identifying influential nodes in complex networks is essential for a wide range of applications, from social network analysis to enhancing infrastructure resilience. While quantum walk-based methods offer potential advantages, existing approaches face challenges in dimensionality, computational efficiency, and accuracy. To address these limitations, this study proposes a novel method inspired by the one-dimensional discrete-time quantum walk (IOQW). This design enables the development of a simplified shift operator that leverages both self-loops and the network’s structural connectivity. Furthermore, degree centrality and path-based features are integrated into the coin operator, enhancing the accuracy and scalability of the IOQW framework. Comparative evaluations against state-of-the-art quantum and classical methods demonstrate that IOQW excels in capturing both local and global topological properties while maintaining a low computational complexity of O(N〈k〉), where 〈k〉 denotes the average degree. These advancements establish IOQW as a powerful and practical tool for influential node identification in complex networks, bridging quantum-inspired techniques with real-world network science applications.

## 1. Introduction

The identification of influential nodes plays a pivotal role across a wide range of domains, including the discovery of critical proteins in protein–protein interaction networks, the selection of product promoters in social media platforms, and the protection of vital nodes in power grids [1,2]. These nodes serve as critical elements within complex networks, and their removal or modification can profoundly impact the system’s vulnerability, security, connectivity, and overall robustness. Despite extensive research, accurately identifying influential nodes remains a challenging and unresolved problem due to the diverse structural roles these nodes may assume across different network contexts.

One common approach to identifying influential nodes involves centrality measures based on neighborhood structure. For example, in social networks, a user with a large number of followers (fans) is often considered a super-spreader. Consequently, the number of direct neighbors can serve as an indicator of a node’s importance within the network [3,4]. This concept can be further extended to multi-hop neighborhoods; for instance, third-degree neighbors are often regarded as the effective range for information diffusion [5]. Neighborhood-based centrality measures are not only limited to counting the number of neighbors but also consider the entropy within the local neighborhood. When a node is regarded together with its surrounding network, similar to how an individual interacts with a complex environment, their mutual influence becomes significant. As a result, factors such as maximal information entropy and the decreasing influence of more distant neighbors are commonly taken into consideration [6,7].

Centrality measures based on neighborhood information are typically classified as local methods. As a result, their performance is generally inferior to that of global methods in identifying influential nodes. For instance, global approaches utilize the complete topological structure of the network. By decomposing the adjacency matrix or the Laplacian matrix, one can extract eigenvalues and eigenvectors that reveal both the importance of individual nodes and the community structures to which they belong [8,9]. Some research evaluates the significance of nodes by analyzing the lengths and frequencies of paths that pass through them [10]. In addition, random walk techniques are widely applied in node mining, where a walker moves from a starting node to its neighbors over a predefined path length until a specified limit is reached. Random walks have inspired numerous node embedding and influential node identification methods, such as DeepWalk [11,12] and approaches based on the quantum counterpart of random walks [13,14]. In some cases, global methods go beyond counting direct connections or neighbors. They assess node importance by progressively removing nodes and observing the resulting network disintegration using robustness indices [15]. Compared to traditional algorithms, such approaches provide more accurate rankings of influential nodes by evaluating their structural impact on the overall network.

Despite the wide range of available information and the abundance of algorithms developed for identifying critical nodes, epidemic dynamic models are often used as standard benchmarks to evaluate algorithm performance. Commonly used models include the Susceptible-Infected-Recovered (SIR), Susceptible-Infected (SI), and Susceptible-Infected-Susceptible (SIS) models [16,17]. Taking the SIR model as an example, each node can be in one of three states: susceptible, infected, or recovered. The influence of a node is measured by the number of nodes it has infected and those that have recovered as a result of its infection. Due to the inherent randomness of the propagation process, the measured values for each node are typically averaged over thousands of independent operations to ensure convergence and obtain reliable, stable estimates.

Additionally, with the growing success of quantum computing in graph analysis and structural mining, several researchers have explored quantum computing to improve the accuracy of influential node identification. Among these efforts, the quantum walk model has attracted considerable attention for its potential advantages. For instance, the classical Google PageRank algorithm has been extended to a quantum version based on Szegedy’s quantum walk [18], marking the beginning of quantum walk techniques being applied to the identification of critical nodes in complex networks. Specifically, in the continuous-time quantum walk model, if the Hamiltonian is replaced by the adjacency matrix of the network or a variant encoding node adjacency, the model can be adapted to both regular and irregular networks [19,20]. This means that the quantum walk model can be applied to any undirected or directed unweighted network, regardless of whether discrete-time or continuous-time quantum walks are employed [21,22]. However, the state space dimension of Szegedy’s quantum walk scales quadratically with the number of nodes in the network, making dimension reduction and accuracy assurance significant challenges for subsequent research. To address this, Liang [23] proposed a quantum walk driven by the Grover operator, with the walk length fixed at three steps, inspired by the three-degree influence rule. This approach reduces the model’s dimension to twice the number of links in the network.

Although the observed results of quantum walks can reflect the structural characteristics of networks, the particle’s movement between nodes during quantum evolution can be disrupted by the quantum interference effect. This implies that the observed outcomes may exhibit chaotic behavior. The repeated traversal of the particle between nodes is referred to as traceback. Minimizing the adverse effects caused by traceback remains a significant challenge in designing methods for identifying influential nodes in complex networks.

In conclusion, improving the accuracy of identifying influential nodes remains a critical task, and there is a continued need to reduce the dimensionality of quantum walk-based models. Consequently, this study proposes a method inspired by the one-dimensional discrete-time quantum walk (IOQW). The main contributions of this study are summarized as follows. (1) In the IOQW method, each node and its direct neighbors are regarded as a whole, with a self-loop added to each node. Thus, the particle starting from any node has two possible directions to move, then the dimensionality of the IOQW is effectively reduced to twice the number of nodes. Furthermore, due to the self-loops, a simplified construction method for the shift operator is discovered, which reduces the complexity of constructing this operator. (2) Self-loops and a step length fixed at two are employed to force the particles to stay at the current node with a higher probability rather than repeatedly tottering between different nodes. In other words, the proposed IOQW mitigates the negative effects of quantum interference during the quantum evolution process. (3) Performance evaluations based on correlation and Kendall coefficient experiments demonstrate that IOQW effectively captures both degree and path characteristics of networks, enabling accurate identification of influential nodes in complex networks.

The remainder of this study is organized as follows. Section 2 defines the one-dimensional discrete-time quantum walk and introduces the innovative IOQW for identifying influential nodes. Section 3 validates the accuracy of the proposed IOQW node discovery by comparing it with well-known quantum methods. Section 4 evaluates the performance of our IOQW against selected non-quantum approaches, using experiments with correlation and the Kendall coefficient to demonstrate its effectiveness in identifying influential nodes in networks.

## 2. IOQW: A Method Inspired by One-Dimensional Discrete-Time Quantum Walk

Existing quantum walk models can be classified into discrete-time and continuous-time types. Discrete-time models integrate structural characteristics of the network into the quantum evolution process, which in turn amplifies the topological characteristics of the network and facilitates the identification of influential nodes. Therefore, this study adopts one-dimensional discrete-time quantum walk (OQW) as the theoretical basis of the proposed IOQW method. By leveraging structural information during the evolution process, the IOQW improves the accuracy of identifying critical nodes. In contrast, due to the constraints imposed by quantum unitary evolution, integrating network features into continuous-time quantum walks on complex networks remains challenging. Therefore, this section begins by introducing the definition of the OQW, which forms the theoretical foundation for the proposed IOQW method. In particular, we explain how one-dimensional discrete-time quantum walks make it possible to reduce the model’s dimensionality to twice the number of nodes.

### 2.1. One-Dimensional Discrete-Time Quantum Walk

Given a one-dimensional line *L*, when a discrete-time quantum walk takes place on *L*, the particle located at an arbitrary node *j* in line *L* has two possible directions: forward $|b\rangle ={\left(0,1\right)}^{T}$ and backward $|f\rangle ={\left(1,0\right)}^{T}$, where T presents the transpose of Dirac vectors. Assuming *L* has *N* nodes, therefore the total number of possible directions is 2N. Consequently, the Hilbert space of the system is defined as $H_{L}=H^{N}⊗H^{2}$, indicating that the dimensionality of the OQW model is 2N.

When defining the orthogonal basis at each node *j* as |j〉, and considering the line *L* as an isolated quantum system whose Hilbert space has dimension 2N, the quantum state $|\psi_{L}\left(0\right)\rangle$ at the initial time can be formulated as

(1) $$|\psi_{L}\left(0\right)\rangle =\sum_{j=1}^{n}\alpha_{j}\left(0\right)|j\rangle ,$$

where |j〉 is a basis vector in an *N*-dimensional Hilbert space, $\alpha_{j}\left(0\right)$ represents the probability amplitude of node *j* at the initial time t=0. For an isolated quantum system *L*, the total probability is conserved during evolution. At any time *t*, the sum of the squared magnitudes of the probability amplitudes satisfies the following condition: $\sum_{j=1}^{n}{\left|\alpha_{j}\left(t\right)\right|}^{2}=1$.

**Figure 1:**
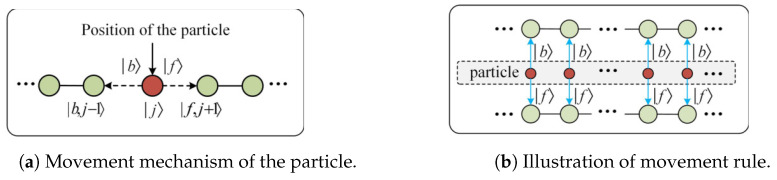
Illustration of particle walking on a line.

Furthermore, since quantum mechanics and quantum computing require the representation of the movement and evolution of quantum states using matrix and Dirac notations, the motion of a particle in the OQW model is represented by the shift operator $S_{L}$, which determines the particle’s movement based on its direction state. For example, the state |b〉 denotes the particle moves from node |j〉 to node |j−1〉, while the state |f〉 corresponds to movement from |j〉 to |j+1〉, as illustrated in Figure 1a. From a quantum perspective, the particle can be viewed as simultaneously moving in both directions defined by |b〉 and |f〉, as reported in Figure 1b. Accordingly, the shift operator $S_{L}$ is defined as follows:

(2) $$S_{L}=\left|f\rangle \langle f\right|⊗\sum_{j}|j+1\rangle \langle j|+|b\rangle \langle b|⊗\sum_{j}|j-1\rangle \langle j|,$$

where notation ⊗ denotes tensor product in quantum mechanics. The OQW model is a type of coined discrete-time quantum walk, where the particle is guided by a coin operator to facilitate movement between nodes. Generally, the coin operator can take various forms depending on the specific application or context, such as Hadamard operator, Grover operator, Fourier operator, or unitary operator from the SU(2) group [23,24,25].

To describe the movement of the particle, the evolution operator $U_{L}$ and corresponding evolution process of |ψ(0)〉 must be constructed. Let the number of walk steps be denoted as *t*, the evolution operator $U_{L}$ is defined as the product of the shift operator $S_{L}$ and the coin operator *C*, i.e.,

(3) $$U_{L}\left(t\right)={\left(S_{L}\cdot C\right)}^{t}.$$

Therefore, by applying Equation (3), the evolution of the quantum state after *t* steps can be expressed as

(4) $$|\psi_{L}\left(t\right)\rangle =U_{L}\left(t\right)|\psi_{L}\left(0\right)\rangle .$$

Finally, after *t* walk steps, the outcome of quantum observation for each node *j* is expressed as a probability $P_{j}\left(t\right)$

(5) $$P_{j}\left(t\right)={\left|\langle j|\psi_{L}\left(t\right)\rangle \right|}^{2}={\left|\langle j\left|U_{L}\left(t\right)\right|\psi_{L}\left(0\right)\rangle \right|}^{2}.$$

In the OQW model, each node on the line *L*, except for the dangling nodes, has two possible directions for the particle to move. Inspired by this mechanism, we propose that in complex networks if each node’s neighbors are treated as an integrated unit and a self-loop is added to each node, then each node can also have two available directions. Based on this idea, the OQW model serves as a foundation for the proposed IOQW method, which effectively reduces the model’s dimensionality.

### 2.2. Definition of the Proposed IOQW

According to Equations (1)∼(5) described in Section 2.1, the definition of the proposed IOQW method can be summarized in four steps, including quantum state, evolution operator, evolution process, and quantum observation. The following sections elaborate on each of these components in detail.

#### 2.2.1. Quantum State of the IOQW

**Figure 2:**
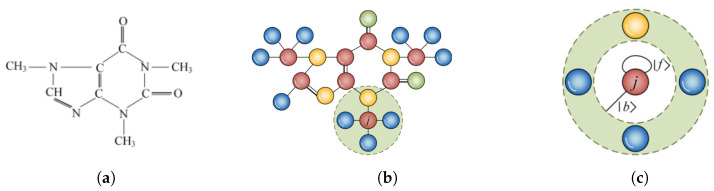
Explanation of complex network and integrated consideration. (**a**) Caffeine molecular. (**b**) Network of Caffeine. (**c**) Integrated neighbors.

Given an undirected network G=(V,E), *V* and *E* denote the node and link sets of *G*, with |V|=N and |E|=M. Consider the caffeine molecule illustrated in Figure 2a as an instance, and its corresponding complex network representation shown in Figure 2b. To define the OQW on network *G*, the neighbors of each node in *G* is required to be compressed. This ensures that there are two possible directions when the particle moves between any pair of nodes. As shown in Figure 2c, if the neighbors of node *j* are treated as an integration and a self-loop is added to node *j*, then *j* has two possible directions, |b〉 and |f〉. With this construction, the OQW model described in Section 2.1 becomes applicable to complex networks. This modification ensures that the state space dimension of the proposed IOQW is 2N, i.e., twice the number of nodes in *G*. Accordingly, considering the orthogonal basis |b〉 and |f〉, the initial quantum state |ψ(0)〉 of IOQW at time t=0 can be defined as follows:

(6) $$|\psi \left(0\right)\rangle =\sum_{c=\{b,f\}}|c\rangle ⊗\sum_{j\in V}\beta_{j}\left(0\right)|j\rangle$$

where $\beta_{j}\left(0\right)$ denotes the probability amplitude of node *j* at t=0. For an undirected network and unweighted network *G*, which can be regarded as an isolated quantum system, the total probability is conserved, and thus it satisfies the normalization condition $\sum_{j\in V}{\left|\beta_{j}\left(0\right)\right|}^{2}=1$. The basis vector |j〉 is defined such that all its elements are zero except for the *j*-th position, which is set to one.

It is worth noting that quantum interference can have both beneficial and detrimental effects. On one hand, it can accelerate the traversal of particles across a network, thereby improving the efficiency of information propagation. On the other hand, it may introduce chaotic behavior due to interference patterns, which complicates quantum measurements. Specifically, quantum interference can cause a particle to repeatedly oscillate between neighboring nodes, a phenomenon known as the traceback effect, leading to unstable or inconsistent measurement outcomes [13,21,23]. To mitigate this effect, self-loops are added to each node in the proposed IOQW model, which increases the likelihood that a particle remains at its current position. This adjustment significantly reduces the impact of traceback and enhances the stability of quantum observation results. Therefore, by incorporating self-loops, the IOQW method achieves more reliable identification of influential nodes in complex networks.

In conventional quantum walks, a common challenge arises when a particle moves between dangling nodes (or leaf nodes) and their neighbors, resulting in limited propagation paths and biased measurements [26]. In contrast, the IOQW method overcomes this limitation by treating each node and its local neighborhood as a whole and assigning a self-loop to every node. As a result, every node provides exactly two possible directions for the particle to evolve, ensuring more consistent and uniform quantum evolution across the entire network.

#### 2.2.2. Evolution Operator of the IOQW

The completed evolution operator of the proposed IOQW consists of a shift operator *S* and a coin operator *C*. Referring to the formulation of the operator defined in the Grover search algorithm [23], we incorporate the path information between any pair of nodes into the Grover operator. As a result, the coin matrix of the IOQW can be defined as follows:

(7) $$C_{j,k}=-A_{j,k}+\frac{2}{Pa(j,k)+2},$$

where Pa(j,k) calculates the shortest path length between node *j* and node *k*, *A* represents the adjacency matrix of *G* and $A_{j,k}$ denotes the link relationship between node *j* and node *k*. Since a longer path indicates weaker influence between nodes, and the minimum value of Pa(j,k) is 1, we add 2 to Pa(j,k) to ensure its value remains below 1. Consequently, the coin operator *C* incorporates both path length and connectivity information, which are used to represent the influence of each node. Based on Equation (7), the final form of the coin operator *C* can be constructed as follows:

(8) $$C=\left[\begin{array}{cccc} C_{1,1} & C_{1,2} & \ldots & C_{1,N}\\ C_{2,1} & C_{2,2} & \ldots & C_{2,N}\\ \vdots & \vdots & \ddots & \vdots \\ C_{N,1} & C_{N,2} & \ldots & C_{N,N} \end{array}\right]\in C^{N\times N}.$$

Research shows that real-world complex networks are often scale-free networks, where a small number of nodes possess a large number of connections [27]. Therefore, the degree distribution is a key indicator of the fundamental structural properties of such networks. In this study, node degree is incorporated as a central feature in the design of the coin operator *C*. It is worth noting that compared to representative quantum walk models on complex networks [26], the complete Grover operator is formed by the direct sum of the sub-operators corresponding to all subsystems. In this study, to identify key nodes in the network, degree centrality is incorporated into the operator *C*, and both the shortest paths and global connectivity are introduced as heuristic information. In Ref. [26], the Grover operator has a dimension of 2M, whereas in the IOQW method, the coin operator *C* has a dimension of 2N. Furthermore, Ref. [26] utilizes phase parameters and Euler’s formula, in conjunction with subsystem decomposition, to construct the Fourier coin operator. However, due to the lack of a clear correlation between the quantum measurement outcomes of phase parameters and topological features of the network, this design approach may be less effective for the task of significant node identification.

The other component of the evolution operator is the shift operator *S*, which governs the motion of the particle. Based on the movement mechanism illustrated in Figure 1, the shift operator *S* is defined as follows:

(9) $$S=\sum_{j\in V}(|f,j\rangle \langle j,f|+\frac{1}{\sqrt{\left|N(j)\right|}}\sum_{k\in N(j)}|b,j\rangle \langle k,b|).$$

where $S\in C^{2N\times 2N}$ due to the tensor product operations.

Although the dimensionality of the proposed IOQW is compressed, the relationships between neighbor nodes are still encoded in the shift operator *S*. From Equation (7)∼(9), the completed evolution operator *U* can be obtained.

(10) $$U=S\cdot \left(C⊗\hat{I}\right),U\in C^{2N,2N}$$

where $\hat{I}$ represents the 2×2 identity matrix. As the coin *C* in Equation (8) has dimension N×N, $\hat{I}\in C^{2\times 2}$ is used to ensure the dimensions of *S* and $\left(C⊗\hat{I}\right)$ are equal.

#### 2.2.3. Evolution Process of IOQW

In quantum algorithms, the evolution process is typically formulated as the multiplication of an evolution operator and an initial quantum state. This computational process is analogous to the core steps in traditional (non-quantum) algorithms for identifying influential nodes. According to Equations (6) and (10), the evolution process of the proposed IOQW can be derived as

(11) $$|\psi \left(t\right)\rangle =U^{t}|\psi \left(0\right)\rangle .$$

Since the proposed IOQW is a discrete-time quantum walk, the parameter *t* denotes a *t*-step walk, indicating that the *t*-hop structural information of network *G* is incorporated into the quantum evolution process. The shift operator *S* captures the global connectivity of *G*, while *t* enhances the local information of *G*. In this study, *t* is set to 2, enabling the proposed IOQW method to simultaneously capture both the global and local features of the network, thereby providing a theoretically more accurate measure of each node’s influence. Many studies have explored the appropriate measurement levels for networks. For example, the three-degree influence principle suggests that effective information propagation only occurs within three degrees, and propagation effects beyond the third degree are extremely weak [23]. In the link prediction, there is a discussion on the impact of 3-hop and 2-hop on the accuracy of link prediction in networks. The conclusion indicates that the accuracy performance of both tends to be similar in most networks. Furthermore, in the design of quantum methods, the traceback effect caused by quantum interference is a double-edged sword. On the one hand, it allows a method to quickly capture the multi-dimensional topological features of a network with parallel advantages. On the other hand, it causes particles to be transferred unpredictably between nodes on the graph. When the walk step *t* is set to 2, the traceback effect caused by quantum interference can be effectively suppressed.

#### 2.2.4. Quantum Observation of IOQW

The quantum observation is used to estimate the influence of each node. The observed outcome of all nodes in *V* is presented as a probability distribution. The estimated value of node *j* can be described as the projection onto the orthogonal basis |j〉 after *t* iterations of quantum evolution. That is, the estimated influence of each node can be represented as:

(12) $$P\left(j\right)=|\langle \hat{j}{\left|U|\psi \left(t\right)\rangle \right|}^{2}={\left|\langle \hat{j}\left|U^{t}\right|\psi \left(0\right)\rangle \right|}^{2}.$$

Then, the probability distribution can be obtained as $P=(P_{1},P_{2},\ldots ,P_{N})$. To guarantee the identification accuracy of the proposed IOQW, the neighbor information N(j) of node *j* is incorporated into the observed outcome. Therefore, the transpose of $\langle \hat{j}|$ can be defined as:

(13) $$|\hat{j}\rangle =\sum_{k\in N(j)}\frac{\sqrt{\left|N(k)\right|}}{M}|k\rangle ⊗\sum_{c=\{b,f\}}|c\rangle$$

where |N(k)| equals to the number of neighbors for node *k*, and $\sum_{c=\{b,f\}}|c\rangle ={\left(1,1\right)}^{T}$.

### 2.3. Optimization and Description of IOQW

Constructing various quantum operators in a quantum algorithm is complex. In the proposed IOQW, the most complex operator, *S*, will be optimized. To be specific, *S* obtained by Equation (9) can be decomposed as $\sum_{j\in V}(|f,j\rangle \langle j,f|$ and $\sum_{j\in V}\frac{1}{\sqrt{\left|N(j)\right|}}\sum_{k\in N(j)}\left|b,j\rangle \langle k,b\right|$. By combining the operations of $|b\rangle ={\left(0,1\right)}^{T}$ and $|f\rangle ={\left(1,0\right)}^{T}$, the following rules can be derived:

(14) $$\sum_{j\in V}(|f,j\rangle \langle j,f|=\left[\begin{array}{cccc} 1 & 0 & \ldots & 0\\ 0 & 1 & \ldots & 0\\ \vdots & \vdots & \ddots & \vdots \\ 0 & 0 & \ldots & 1 \end{array}\right]=I\in C^{N\times N},$$

(15) $$\sum_{j\in V}\frac{1}{\sqrt{\left|N(j)\right|}}\sum_{k\in N(j)}\left|b,j\rangle \langle k,b\right|=\sum_{j=1}^{N}\frac{1}{\sqrt{\left|N(j)\right|}}A\left[j\right].$$

For simplicity, the result obtained from Equation (15) is denoted as $\hat{A}$. Merging Equations (14) and (15), the construction process of the operator *S* can be simplified:

(16) $$S=\left[\begin{array}{cc} \hat{1} & 0\\ 0 & \hat{A} \end{array}\right]=\hat{1}⊕\hat{A},$$

where notation ⊕ denotes direct sum operation, if a matrix $A\in C^{N\times N}$ and a matrix $B\in C^{2\times 2}$ perform the ⊕ operation, then a matrix $C\in C^{2N\times 2N}$ can be obtained. To optimize this, the computational complexity is decreased, and since the dimension of the proposed IOQW on a complex network is reduced to 2N, the identification efficiency of influential nodes using the proposed IOQW can be improved.

To describe the proposed IOQW method, the pseudocode are given in Algorithm 1. In the input stage, an empty matrix $\hat{A}\in C^{N\times N}$, an all-ones vector vecOnes with dimension *N*, and an identity matrix $idenM\in C^{N\times N}$ are initialized. Lines 1 and 2 are used to obtain the adjacency matrix of network *G* and to initialize quantum state, respectively. From lines 3∼8, the coin operator *C* and the part of shift operator *S* are constructed based on Equations (7)∼(9) and (15). Furthermore, the completed *S* and evolution operator *U* after *t*-steps are described in lines 9 and 10, where $\hat{I}$ is an identity matrix with dimension 2×2. Additionally, the quantum observation process is exhibited according to Equations (12)∼(13) from lines 11∼18, where temp is an empty vector with a dimension *N*. Finally, the influence score of each node is recorded in distribution *P*, and the outcomes, sorted in decreasing order, are returned, as shown in line 19.

Since the neighbors of each node are visited one by one, the time complexity is O(N〈k〉) for both lines 3∼8 and lines 11∼18, where 〈k〉 denotes the average degree. In summary, the time complexity of the proposed IOQW equals O(N〈k〉+N〈k〉) and the maximum dimension of matrices used in IOQW is 2N×2N.

In summary, the advantages of the proposed IOQW method can be outlined as follows. (i) The complexity of the shift operator in the proposed IOQW is easier to implement compared to other discrete-time quantum walks. (ii) The IOQW incorporates diverse structural information during both the quantum evolution process and quantum observation, which enhances the identification accuracy in identifying influential nodes. (iii) With a low-dimensional representation of 2N, the efficiency of the IOQW for finding influential nodes is relatively improved. (iv) By setting the walk step to 2, the method avoids the need to reach convergence, thereby reducing the number of required operations.
**Algorithm 1** IOQW for identifying influential nodes in *G***Require:** $G=\left(V,E\right),t,\hat{A},vecOnes,identM$  1:Adj=getGraph(G);  2:$\psi ⇐\frac{1}{\sqrt{N}}vecOnes;$  3:**for** each *j* in *V* **do**:  4:      $\hat{A}\left[j\right]⇐\frac{1}{\sqrt{\left|N(j)\right|}}\hat{A}\left[j\right];$  5:      **for** each *k* in N(j) **do**:  6:            $C_{j,k}⇐-Adj_{j,k}+\frac{2}{Path(j,k)+2};$  7:      **end for**  8:**end for**  9:$S⇐identM⊕\hat{A}$;10:$U\left(t\right)⇐{\left[S\left(C⊗\hat{I}\right)\right]}^{t};$11:**for** each *j* in *V* **do**:12:      temp⇐[];13:      **for** each *k* in N(j) **do**:14:            $temp\left[k\right]⇐\frac{\sqrt{\left|N(k)\right|}}{M}⊗|k\rangle ;$15:      **end for**16:      temp⇐temp∪temp;17:      $P\left(j\right)⇐{\left(\langle temp|U\left(t\right)|\psi \rangle \right)}^{2};$18:**end for**19:**return** sorted(*P*) by decreasing;

## 3. Comparison to Quantum Methods

Due to the computational complexity involving tensor products, exponential terms with negative exponents, and the expansion of matrix dimensions, many quantum algorithms for identifying influential nodes are typically limited to small-scale networks when run on classical computational devices. Therefore, this section evaluates the performance of the proposed IOQW method by comparing it with other quantum algorithms in terms of their ability to identify influential nodes.

### 3.1. Theoretical Comparison and Analysis with IOQW

Since quantum walks are regarded as the quantum counterpart of conventional random walks [28], this section compares the proposed IOQW method with the conventional random walk and other effective quantum algorithms, including quantum PageRank (QPageRank) [18], Grover walk (GWalk) [23], quantum centrality (QCent) [19], continuous-time quantum walk-based information propagation model (CQIPM) [22], and coined quantum walk for community detection (CQW) [26]. In the above methods, the QPageRank is extended from the well-known Szegedy’s quantum walk, which is inspired by the Google probability transition matrix to identify significant nodes. QCent uses a continuous-time quantum walk with the network’s adjacency matrix as the Hamiltonian and computes the average over an infinite number of quantum evolutions. Also based on continuous-time quantum walks, the CQIPM model incorporates propagation features to measure node influence. The GWalk method applies a three-degree influence rule with a walking step of 3 to discover node importance. Additionally, CQW treats the nodes and their direct neighbors as subsystems, constructing the complete Hilbert space through the composite of all systems and thereby defining the discrete-time quantum walk on a complex network. CQW defines two different coin operators, namely the Grover and Fourier operators, and since its application field is community detection, further discussion will not be provided.

Table 1 provides details of types, dimensions, walk steps, and heuristic information incorporated for each method. In the table, Shift represents the construction method of the shift operator, State records the heuristic information embedded in different state vectors, while Evolution and Observation indicate the network feature information incorporated during the quantum evolution and measurement processes, respectively. It is worth noting the matrix ϕ and Λ denote the ordered eigenvectors and the diagonal matrix of the ordered eigenvalues of ϕ.

To enhance the identification accuracy, various heuristic information is incorporated into quantum methods. The following conclusions can be summarized. (i) Incorporating heuristic information into the initial quantum state is challenging, and the dimensionality increases with the addition of beneficial information. (ii) The proposed IOQW method features a simpler shift operator compared to other methods. However, continuous-time quantum walks involve the negative exponential power operation of *e*, resulting in generally higher time complexity than others. Moreover, the extreme dimensionality of QPageRank makes it difficult to apply in larger networks. (iii) A quantum method performing a *t*-step walk includes *t*-hop structural information in the quantum evolution process. In methods like QCent and NCCQ (node centrality based on continuous-time quantum walk) [29], setting *t* to infinity can lead to chaotic characteristics in heuristic information, negatively affecting the identification of influential nodes. In contrast, the proposed IOQW method uses only 2-step walks, which efficiently captures global connectivity while avoiding excessive complexity. Research has shown that local topological information, such as 2-hop details, effectively reflects network features in various applications, such as link prediction [21,30] and community detection [31].

### 3.2. Study Case Using Various Quantum Algorithms

**Figure 3:**
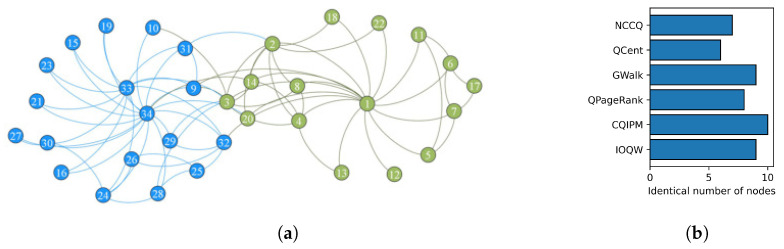
Illustration of the Karate network and compared results. (**a**) Visualization of the Karate network. (**b**) Compared to PageRank with the same nodes.

To compare the effectiveness of IOQW and other quantum methods in identifying influential nodes, taking Zachary’s Karate Club network as an example, which consists of 34 nodes and 78 links. Two prominent members are nodes 1 and 34. As illustrated in Figure 3, the network is divided into two communities, with leader nodes 1 and 34 highlighted in blue and green, respectively. Many quantum walk methods are extremely computationally intensive to simulate on current von Neumann architecture-based computers, especially on networks with more than 100 nodes. Therefore, smaller open-source networks are selected for testing. To assess identification accuracy, Table 2 presents the computational results for the top-10 influential nodes ranked by various quantum methods introduced in Section 3.1, where to ensure a fair comparison, the ranking results of the classical PageRank algorithm are introduced as a baseline for the quantum methods.

As shown in Table 2, the IOQW method identifies 9 nodes, CQIPM 10 nodes, QPageRank 8 nodes, GWalk 9 nodes, QCent 6 nodes, and NCCQ 7 nodes among the top-10 influential nodes, in comparison to the PageRank algorithm. Figure 3b supports this comparison. The IOQW method effectively incorporates degree and path heuristic information, aligning closely with the PageRank results. It accurately identifies high-degree nodes like 1, 33, 34, 32, and 4, as well as hub nodes 9, 3, and 14 bridging communities. Notably, IOQW also identifies nodes 8 and 9, which lack obvious structural features, demonstrating its superior identification capability over other quantum algorithms.

## 4. Comparison to Traditional Methods

This section chooses the selected algorithm for identifying influential nodes as a comparison, including degree centrality [32], Betweenness centrality (BC) [32], centrality degree paths (CDP) [33], distance Laplacian centrality (DLC) [34], and isolating betweenness centrality (ISBC) [35]. In the compared methods, Degree and BC are commonly used metrics in network analysis tools such as Gephi 0.10 and Python 3.9 libraries like NetworkX. The CDP method calculates node influence based on degree and path lengths, DLC focuses on network density, and ISBC integrates both local and global structural information to assess influence. To compare the efficiency of execution, Table 3 presents the time complexity of the proposed IOQW alongside other methods, where *M* and 〈k〉 denote the number of links and the average degree of the network, respectively. Notably, the IOQW method requires fewer computational resources than BC, DLC, and ISBC approaches.

Furthermore, the six complex networks are selected as experimental datasets to evaluate the accuracy of different methods. Their statistical characteristics are summarized in Table 4, where 〈k〉, $d_{max}$, and *D* represent the average degree, maximum degree, and diameter of the network, respectively. In addition, the notation *c* and *a* present the cluster coefficient and assortativity coefficient, respectively.

### 4.1. Experiment on Correlation Between SIR and Various Methods

In the identification of influential nodes, the Susceptible-Infected-Recovered (SIR) model [36] is considered a reliable criterion to estimate the identification accuracy of an algorithm. An arbitrary node in the SIR model is in one of three states: susceptible, infected, or recovered, the number of infected nodes and recovered nodes is represented by the number of infected and recovered nodes, referred to as the F(t) value. Since the propagation process has randomness, the F(t) of each node is obtained by performing $10^{3}$ independent simulations. To satisfy the outbreak threshold, the propagation probability *p* between a pair of nodes is set to different values across networks according to $p\geq \frac{\langle k\rangle }{\langle k^{2}\rangle -\langle k\rangle }$, where $\langle k^{2}\rangle$ is the second moment of the degree distribution of the network.

**Figure 4:**
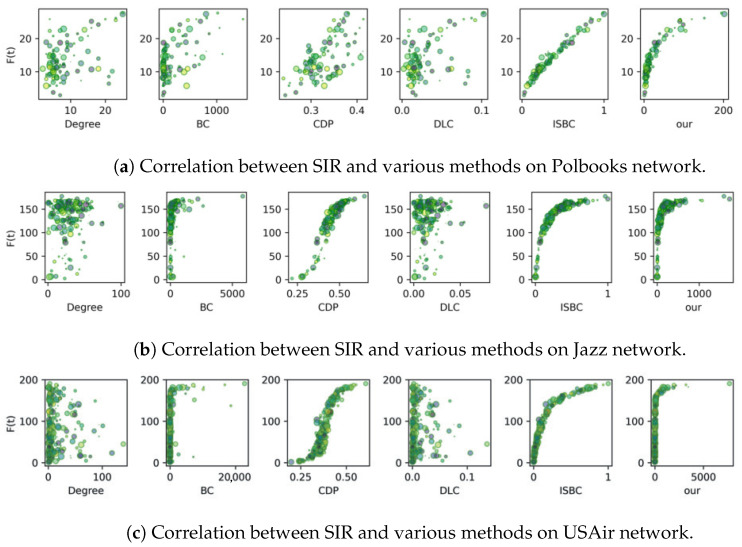
Correlation between SIR and various methods on Polbooks, Jazz, and USAir networks.

**Figure 5:**
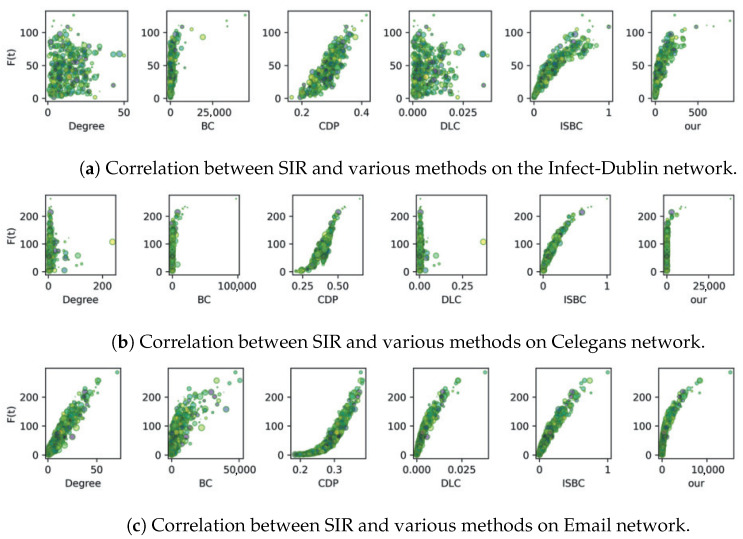
Correlation between SIR and various methods on Infect-Dublin, Celegans, and Email networks.

The correlation experiment involves generating scatter plots that compare the node influence values computed by various methods with those derived from the SIR model. Figure 4 and Figure 5 display the results for six complex networks, where each scatter point is visualized with randomized colors and sizes. A strong correlation is reflected by a clear trend or curve in the plots, indicating the degree of consistency between the algorithm outputs and the SIR-based evaluations.

As reported in Figure 4a, the proposed IOQW and ISBC exhibit stronger and more consistent correlations with the SIR model compared to other methods on the Polbooks network. On the Jazz and USAir networks, the measured values using CDP, ISBC, and the proposed IOQW align closely with the F(t) value of the SIR model. In contrast, as depicted in Figure 4b,c, the Degree and DLC methods exhibit more erratic patterns, suggesting that these techniques—particularly the simplistic degree method and the matrix decomposition-based DLC—are less robust in identifying influential nodes.

The analysis in Figure 5 reveals that while most comparison algorithms perform moderately under the SIR model, the ISBC and the proposed IOQW methods stand out with superior correlations. Figure 5a highlights that both IOQW and ISBC outperform other algorithms. On the Celegans network, as illustrated in Figure 5c confirms that the IOQW method exhibits the strongest correlation with the SIR model among all evaluated algorithms.

Overall, based on the comprehensive analysis of Figure 4 and Figure 5, the Degree and DLP methods consistently underperform in identifying influential nodes under the SIR model. In contrast, the proposed IOQW and ISBC methods exhibit outstanding and consistent performance across various complex networks, highlighting their robustness and accuracy in influence identification.

### 4.2. Experiment on Kendall Coefficients Between Different Methods

Kendall coefficient τ is used to quantify the correlation between measured results of two algorithms, especially when an algorithm is applied to identify significant nodes in a complex network. Given two measured results, *X* and *Y*, suppose a pair of measured values are denoted as $\left(x_{j},y_{j}\right)$ and $\left(x_{k},y_{k}\right)$. The Kendall coefficient τ is then defined as:

(17) $$\tau =\frac{N_{c}-N_{d}}{\frac{1}{2}N\left(N-1\right)},$$

where $N_{c}$ and $N_{d}$ represent the amount of positive correlation and the amount of negative correlation, respectively. When the relationship between $x_{j}$ and $x_{k}$, between $y_{j}$ and $y_{k}$ satisfy the following conditions, the $N_{c}$ and $N_{d}$ are defined as

(18) $$\left\{\begin{array}{c} N_{c}=N_{c}+1,\left[\left(x_{j}>x_{k}\right)\cap \left(y_{j}>y_{k}\right)\right]\cup \left[\left(x_{j}<x_{k}\right)\cap \left(y_{j}<y_{k}\right)\right]=T\\ N_{d}=N_{d}+1,\left[\left(x_{j}<x_{k}\right)\cap \left(y_{j}>y_{k}\right)\right]\cup \left[\left(x_{j}>x_{k}\right)\cap \left(y_{j}<y_{k}\right)\right]=T \end{array}.\right.$$

**Figure 6:**
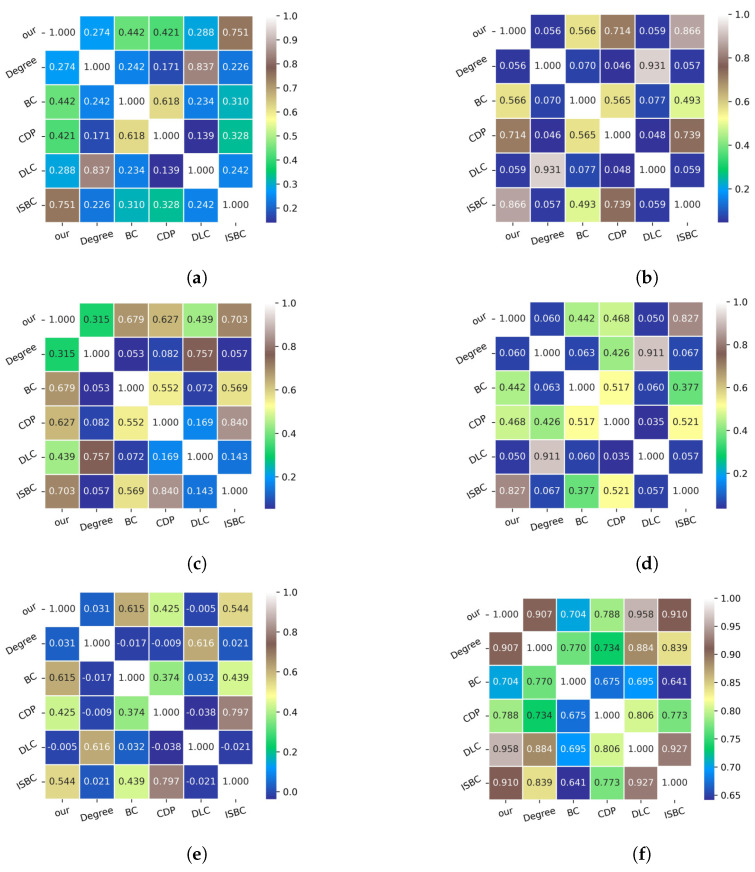
Explanation of complex network and integrated consideration. (**a**) Kendall coefficient on Polbooks network. (**b**) Kendall coefficient on Jazz network. (**c**) Kendall coefficient on USAir network. (**d**) Kendall coefficient on Infect-Dublin network. (**e**) Kendall coefficient on Celegans network. (**f**) Kendall coefficient on Email network.

In Equation (18), T denotes the condition established. Generally, the value obtained by Equation (17) belongs to [−1,1], where −1 indicates no correlation between set *X* and *Y*, and 1 demonstrates a perfect correlation. The experiment on the Kendall coefficient between various methods is depicted in Figure 6.

Both the ISBC and the proposed IOQW methods incorporate features related to the shortest path and degree. BC is a representative index for path measurement. The average Kendall coefficient τ between BC and ISBC on the six networks is 0.472, calculated as $\frac{1}{6}\times \left[0.310+0.493+0.569+0.377+0.439+0.641\right]$. However, the average τ between BC and the proposed IOQW is higher, at 0.574. In comparison, the average τ between DLC and BC of which are path counting indices, is relatively low at 0.195. Additionally, the average Kendall coefficient between Degree and ISBC is 0.211, while it is higher at 0.274 between Degree and IOQW. Consequently, the average τ between ISBC and IOQW across the six networks reaches 0.766.

Combining Figure 4 with Figure 5a,b, it is evident that the degree performs poorly in identifying influential nodes under the SIR model. Specifically, the Kendall coefficient τ between Degree and BC, as well as between Degree and CPD, between Degree and ISBC, is notably low, as illustrated in Figure 6a–c,e. This indicates weak correlations and underscores the limitations of the degree metric in effectively capturing node influence.

Overall, the proposed IOQW method captures both degree-based and path-based characteristics effectively, demonstrating its strong capability to identify influential nodes in complex networks.

## 5. Conclusions

To address the challenges posed by quantum interference and the high dimensionality of quantum walks on complex networks, this study proposed a method inspired by the one-dimensional quantum walk for identifying influential nodes, referred to as IOQW. The proposed IOQW method reduced the dimensionality of the state vector to 2N, twice the number of nodes, by introducing self-loops and integrating neighborhood structures. This design simplifies the quantum model while enhancing the identification of influential nodes, thereby improving both accuracy and computational efficiency. Experimental results based on correlation analysis demonstrated that the proposed IOQW method consistently outperforms existing techniques in identifying influential nodes across 6 real complex networks. Furthermore, the Kendall coefficient evaluations confirmed that IOQW effectively captures both degree and path structural features, validating its robustness and capability in representing critical network characteristics.

There are several challenges associated with applying quantum walks to the task of identifying influential nodes in complex networks. This study specifically focuses on undirected and unweighted complex networks; however, future research could extend quantum walk models to more diverse network types, including multi-layer, directed, and temporal networks. Each of these network types introduces unique challenges. For instance, in directed networks, the corresponding quantum system operates as an open quantum system, making the definition and implementation of quantum particle dynamics considerably more complex.

In addition, testing on synthetic networks is essential to rigorously verify the theoretical time complexity of O(N〈k〉). Future studies should evaluate the scalability of the proposed IOQW method across synthetic networks of varying sizes, including (a) ER networks and (b) scale-free networks with controllable degree exponents. Moreover, quantifying the influence of self-loop weights on the stability of quantum interference will further strengthen the theoretical foundation of the IOQW method. These future directions are critical for validating IOQW’s robustness and scalability and for ensuring its applicability to large-scale, highly heterogeneous network environments.

## Figures and Tables

**Table 1:** Compared with various quantum methods.

	IOQW	QPageRank	GWalk	QCent	CQIPM	CQW
Types	DT	DT	DT	CT	CT	DT
Dimensions	2N	$N^{2}$	2M	*N*	*N*	2M
*t*-steps	2	50	3	Infinite	0.1	100
Shift	$S=\hat{1}⊕\hat{A}$	$S=\sum_{j\in V}^{k\in N(j)}\left|j,k\rangle \langle k,j\right|$	S|j,k〉=|k,j〉	$U=e^{-iAt}$	$U=e^{-iAt}$	S|j,k〉=|k,j〉
State	None	Probability matrix	Degrees	None	Degree	Degree
Evolution	Path	Degree	Connectivity	Connectivity	Connectivity	Phase or degree
Observation	Degree	None	Diffusion feature	None	None	Degree

**Table 2:** Influential nodes ranked by various quantum methods.

	IOQW	PageRank	CQIPM	QPageRank	GWalk	QCent	NCCQ
Top-1	1	34	34	34	1	34	1
Top-2	34	1	1	1	34	2	34
Top-3	3	33	33	33	3	1	3
Top-4	33	3	3	2	33	28	4
Top-5	2	2	2	3	2	3	14
Top-6	14	32	4	32	14	27	33
Top-7	4	4	32	4	32	20	9
Top-8	8	24	14	6	9	14	8
Top-9	9	9	9	7	4	4	20
Top-10	32	14	24	24	31	26	26

**Table 3:** The time complexity of compared methods.

	Degree	BC	CDP	DLC	ISBC	IOQW
Time complexity	O(M)	$O(N^{3})$	O(M+N〈k〉)	$O(N^{3})$	$O(N^{2}+N\langle k\rangle )$	O(2N〈k〉)

**Table 4:** Statistical characteristics of complex networks.

	*N*	*M*	〈k〉	$d_{\max}$	*D*	*c*	*a*
Polbooks	105	441	8.400	25	7	0.488	−0.475
Jazz	198	2742	27.697	100	6	0.618	0.020
USAir	332	2126	12.807	139	6	0.039	−0.207
Infect-Dublin	410	2765	13.488	50	9	0.467	0.225
Celegans	453	2025	8.940	237	7	0.655	−0.225
Email	1133	5451	9.622	71	8	0.22	0.078

## Data Availability

The open-source complex network datasets used in this paper can be downloaded from https://networkrepository.com/.
